# Dyadic Associations of Parenting Stress, Family Resilience, and Coping Styles Among Parents and Grandparents in Intergenerational Co-Parenting Families

**DOI:** 10.1155/nrp/8036580

**Published:** 2025-07-11

**Authors:** Juanjuan Ma, Dan Chen, Kaiyue Wang, Chaonan Li, Lining Wang, Hui Zhang

**Affiliations:** ^1^Basic Nursing Department, School of Nursing, Harbin Medical University, Daqing, Heilongjiang, China; ^2^Nursing Department, Xing'an Vocational and Technical College, Ulanhot, Inner Mongolia, China; ^3^Maternity and Gynecological Department, Daqing Oilfield General Hospital, Daqing, Heilongjiang, China; ^4^Liming Community Health Service Center of Daqing, Daqing People's Hospital, Daqing, Heilongjiang, China

**Keywords:** Actor-Partner Interdependence Mediation Model (APIMeM), coping strategies, family resilience, intergenerational co-parenting, parenting stress

## Abstract

**Aim:** This study examined the dyadic relationships among parenting stress, family resilience, and coping styles within Chinese intergenerational co-parenting households.

**Design:** A cross-sectional study was conducted in China from December 2022 to September 2023. The STROBE checklist was used to report the current study.

**Methods:** A total of 312 parent-grandparent dyads caring for children under three years old participated. Both parents and grandparents completed the Parenting Stress Scale, Grandparenting Parenting Stress Scale, Family Hardiness Index, and Simplified Coping Style Questionnaire. Dyadic associations were analyzed using the Actor-Partner Interdependence Mediation Model (APIMeM) to examine whether parenting stress was associated with family resilience and coping styles among parents and grandparents.

**Results:** Family resilience was found to partially mediate the relationship between parenting stress and positive coping styles in both parents (*β* = −0.054, *p* < 0.001) and grandparents (*β* = −0.067, *p* < 0.001). Additionally, family resilience mediated partner effects, suggesting interdependence between parents' (*β* = −0.311, *p* < 0.001) and grandparents' stress and coping mechanisms (*β* = −0.231, *p* < 0.001).

**Conclusions:** This study enhances understanding of the interdependent relationships between parenting stress, family resilience, and coping strategies in Chinese intergenerational co-parenting households.

**Summary**

• Impact: Nurses should consider implementing dyadic interventions aimed at strengthening family resilience, which may improve coping ability and family functioning in intergenerational caregiving families.

## 1. Introduction

In China, the fast-paced modern life and intense competition put parents of infants and young children under significant work and life pressure, leaving them with limited time and energy for childcare [1]. Notably, caring for children under 3 years old demands full-time and long-term attention from caregivers. Consequently, the involvement of grandparents in raising grandchildren is a common occurrence in China [2]. A recent survey report showed that 48.4% of grandparents were involved in rearing grandchildren, and it also reached up to 72.9% in urban households which is much higher than in the rural areas [3]. Additionally, the number of mutigenerational households is also on the rising across China.

Co-parenting has two sides. The care support provided by grandparents can alleviate parental stress, which is beneficial for the physical and mental health of parents and also exerts a positive influence on children's development [4, 5]. Some studies indicated that co-parenting could also enhance the quality of life for grandparents, prevent cognitive function decline and depression, and increase physical activity, potentially reducing the risk of diseases [6–8]. However, parenting stress is common in intergenerational co-parenting families and has a negative impact on the psychological well-being of parents, children, and grandparents [9, 10]. Tan et al. investigated 332 intergenerational co-parenting households and found that mother's parenting stress was at a medium level [11]. Ross et al. also pointed out that 94% of grandparents experienced a high level of parenting stress when caring for children aged 1–3 years old [12]. The findings of Buckingham-Howes et al. showed that conflicts between mothers and mothers-in-law during the first 24 months of a child's life could predict the elevated levels of externalizing behaviors in children at 7 years old [13].

Family Systems Theory emphasizes the interdependence of family members, justifying the use of dyadic analysis to explore how stress and coping strategies in one caregiver impact the other [2, 14, 15]. In intergenerational co-parenting households, when facing parenting stress, caregivers with different characteristics adopt diverse coping styles [16]. Some families are inclined to employ positive coping styles, such as actively solving problems, seeking solutions, and communicating effectively. This approach can contribute to the enhancement of family function and intimacy, and the opposite is also true [17]. Parenting stress can influence parenting coping styles, and the extent of this effect may differ depending on an individual's psychological resilience. The ABC-X model also posits that resources and cognition can mediate the relationship between stress and outcomes [18]. When individuals encounter stressful events, they can assess the situation, mobilize their internal and external resources, formulate coping strategies, and take appropriate actions [2]. In other words, family resilience as the resources in the intergenerational families can buffer the impact of parenting stress, which in turn, can affect the coping styles of parents and grandparents. Numerous studies have demonstrated a significant negative correlation between family resilience and parenting stress. Specifically, the higher the level of family resilience, the lower the perceived parenting stress among parents [19–21].

Thus, based on the literature and theoretical frameworks, we hypothesize that (1) higher levels of parenting stress will be associated with lower levels of family resilience and more negative coping strategies for both parents and grandparents (actor effects); (2) parenting stress in one caregiver will be associated with coping strategies and resilience in the other caregiver (partner effects); and (3) family resilience will mediate these associations.

## 2. Materials and Methods

### 2.1. Aims

1. To investigate the levels of parenting stress, family resilience, and coping styles among parents and grandparents in intergenerational co-parenting families, as well as the relationships among these variables.
2. To examine the Actor-Partner Interdependence Mediation effect of family resilience between parents and grandparents in relation to parenting stress and coping styles.

**Figure 1:**
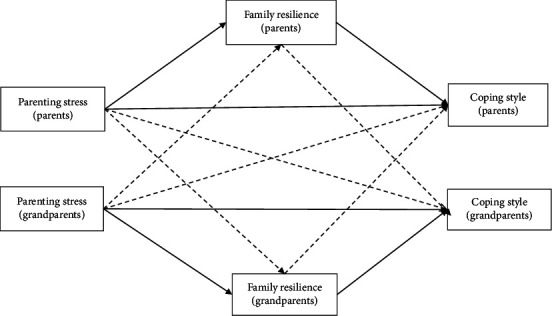
Hypothesized relations among parents' and grandparents' parenting stress, family resilience, and coping style relationships.

The hypothesis model for this study is depicted in Figure 1.

### 2.2. Design

A cross-sectional study was conducted from December 2022 to September 2023 at a Community Health Service Center in Daqing, Heilongjiang Province, which is located in the northeast of China. The current study explored the Actor-Partner Interdependence Mediating roles of family resilience in the relationship between parenting stress and coping styles within Chinese intergenerational co-parenting families with children under 3 years old.

### 2.3. Study Setting and Sampling

A convenient sample of parents and grandparents was recruited from the children's healthcare department of the Liming Community Health Service Center, where approximately 3000 infants are born annually. We approached potential parents and grandparents when their children were undergoing health check-ups or vaccinations and then invited them to participate in the study. The inclusion criteria were as follows: (a) children aged 0–36 months; (b) parents living with at least one grandparent and jointly caring for the children; (c) individuals serving as primary caregivers, providing no less than 30 h of childcare per week [22]; and (d) those willing to participate in this survey. Parents and grandparents with a history of severe mental or physical illness were excluded.

Based on the principle that the ratio of the sample size to the number of observed variables should be at least 10:1, and given that a structural equation model typically requires a minimum of 200–500 samples [23], our study had a total of 18 variables, encompassing both sociodemographic and psychological data. Thus, the minimum sample size for this study was calculated to be 180–270 pairs. Taking into account an estimated 20% invalid responses, we expanded the minimum sample size to 216–324 pairs.

### 2.4. Data Collection

Parents and grandparents who met the inclusive criteria were invited to participate in this survey. The first author explained the aims and details of the study to them. To prevent mutual influence, they were invited to separate quiet rooms and asked to understand and complete the questionnaire independently. All information was guaranteed to be kept confidential. The data were collected by the authors (J.J. and L.N.). Completing the questionnaire took 10–15 min. Verbal consent was obtained from all participants prior to the survey. A standardized written script detailing the research purpose, potential risks, benefits, and participants' right to withdraw was consistently delivered via audio recording to ensure uniform information provision to all participants. After the survey, all participants received a small gift for their children as a token of appreciation. This study followed the guidelines for human studies and was carried out based on the World Medical Association Declaration of Helsinki. This study was approved by the Ethics Committee of Harbin Medical University of Daqing (No. HMUDQ20240422003). A total of 334 pairs of questionnaires were distributed, and 312 pairs were effectively retrieved in this study, resulting in an effective response rate of 93.4%.

### 2.5. Instruments

#### 2.5.1. Sociodemographic Information

The sociodemographic information of parents and grandparents included age, educational level, occupation, per capita monthly household income, and the length of time they had been caring for the child. Additionally, information regarding the child, such as age in months, gender, and birth order, was also collected.

#### 2.5.2. Parenting Stress Scale (PSS)

The PSS, developed by Jones and Berry in 1995, measures parents' perceptions and stress associated with raising children [24]. Cheung translated the scale into Chinese, and it has demonstrated good psychometric properties among parents [25]. This 17-item scale consists of two dimensions: burden pressure and parental role satisfaction. Each item is rated on a five-point Likert scale (1 = strongly disagree, 5 = strongly agree). The total score ranges from 5 to 85, with higher scores indicating a greater perception of parenting stress. In this study, the Cronbach's α for parents was 0.758. Meanwhile, the Grandparents' PSS (GPSS), which revised by Zhang based on the PSS, measures grandparents' perceptions and stress related to caring for grandchildren [26]. The items and scoring system are similar to those of the PSS. In this study, the Cronbach's α for grandparents was 0.883.

#### 2.5.3. Family Hardiness Index (FHI)

The FHI was developed by McCubbin in 1996. It measures family resilience and has been widely used by family members [27]. The FHI consists of 20 items and is composed of three subscales: responsibility, control, and challenge. Each item is rated on a four-point Likert scale (1 = strongly disagree, 4 = strongly agree). The total score ranges from 20 to 80, with higher scores indicating greater family resilience. In this study, the Cronbach's α values were 0.792 for parents and 0.851 for grandparents, respectively.

#### 2.5.4. Simplified Coping Style Questionnaire (SCSQ)

The SCSQ was developed by Xie in 1998 to measure coping styles [28]. The SCSQ consists of 20 items; it is divided into two subscales: the positive coping style and the negative coping style. Each item is rated on a four-point Likert scale (0 = not at all, 3 = often). The total score for the positive coping style ranges from 0 to 36, while that for the negative coping style ranges from 0 to 24. In this study, the Cronbach's α values were 0.90 for parents and 0.725 for grandparents, respectively.

### 2.6. Ethical Statement

This study was approved by the Ethics Committee of the Harbin Medical University (Daqing) (HMUDQ20240422003). The research adhered to the Declaration of Helsinki.

### 2.7. Data Analysis

Data analyses were performed using IBM SPSS 25.0 for Windows and AMOS 24.0. Descriptive statistics were calculated for sociodemographic characteristics, child-related information, parenting stress, family resilience, and coping styles (both positive and negative) of parents and grandparents. Paired-sample *t* tests and *χ*^2^ tests were employed to compare differences in parenting stress, family resilience, and coping styles between parents and grandparents. Pearson's correlation analysis was utilized to identify relationships among these variables. Subsequently, the Actor-Partner Interdependence Mediation Model (APIMeM) was examined using structural equation modeling. Following the guidance of Ledermann et al. [29], all variables were standardized prior to analysis. Bias-corrected bootstrapped 95% confidence intervals (CIs) were used to test actor and partner effects. Model-fit assessment indicators included the root-mean-square error of approximation (RMSEA < 0.05), comparative fit index (CFI > 0.90), adjusted goodness-of-fit index (AGFI > 0.90), root-mean-square residual (RMR), and the *χ*^2^/degrees of freedom (df) ratio (*χ*^2^/df < 3). The dataset used and/or analyzed during this study is available from the corresponding authors upon reasonable request.

## 3. Results

### 3.1. Characteristics of Participants

In this study, a total of 312 pairs of parents and grandparents were surveyed. The mean age of the children was (14.32 ± 9.52) months. The mean ages of the parents and grandparents were (31.72 ± 3.77) years and (59.18 ± 2.96) years, respectively, as presented in Table 1.

### 3.2. Comparison of Parenting Stress, Family Resilience, and Coping Style Between Parents and Grandparents

As presented in Table 2, parents exhibited more severe parenting stress, along with higher scores in family resilience and positive coping, when compared to grandparents (*p* < 0.001). Conversely, grandparents had higher scores in negative coping than parents (*p* < 0.001).

### 3.3. The Correlations Among Parenting Stress, Family Resilience, and Coping Style in Parents and Grandparents

In intergenerational co-parenting families, positive coping was negatively correlated with both parenting stress and family resilience. Negative coping, on the other hand, was positively correlated with parenting stress and negatively correlated with family resilience. Additionally, parenting stress was negatively correlated with family resilience. Moreover, significant correlations were observed between parenting stress, family resilience, and coping style across parents and grandparents (*p* < 0.01). The details are presented in Table 3.

### 3.4. Dyadic Effects Among Parenting Stress, Family Resilience, and Coping Style

The data fit of the APIMeM for the relationships among parenting stress, family resilience, and positive coping style was satisfactory (GFI = 0.981, AGFI = 0.914, *χ*^2^*/*df = 1.754, RMR = 0.039, RMSEA = 0.047). For both parents and grandparents, higher levels of parenting stress were significantly associated with lower levels of family resilience and positive coping style. Conversely, higher levels of family resilience were significantly associated with higher levels of positive coping style (see Table 4).

**Figure 2:**
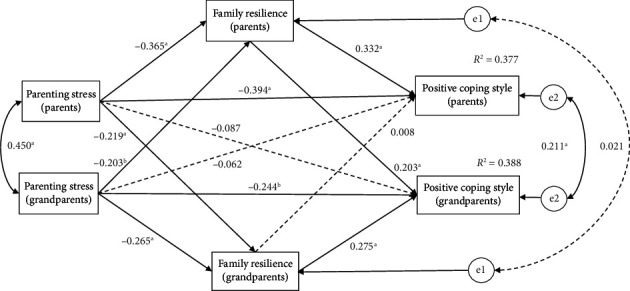
The Actor-Partner Interdependence Mediation Model of parenting stress and family resilience on positive coping style. Note: ^a^*p* < 0.001, ^b^*p* < 0.01, ^c^*p* < 0.05.

The results of APIMeM indicated that family resilience partially mediated the relationship between parenting stress and positive coping styles in both parents (*β* = −0.394, *p* < 0.001) and grandparents (*β* = −0.244, *p* < 0.001), thus supporting the hypothesis of this study. Moreover, the family resilience of parents played a completely mediating role in the relationship between parenting stress in parents and positive coping styles in grandparents (*β* = −0.073, *p* < 0.001), which partially supported the research hypothesis (see Table 4 and Figure 2).

For both parents and grandparents, a significant association was found between higher parenting stress and lower family resilience, as well as between higher parenting stress and a more pronounced negative coping style. Additionally, there was a significant correlation between lower family resilience and a higher negative coping style (see Table 5).

**Figure 3:**
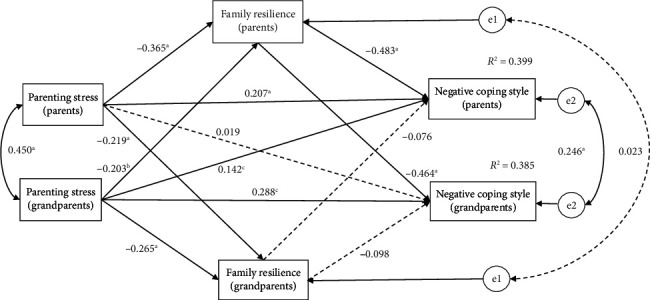
The Actor-Partner Interdependence Mediation Model of parenting stress and family resilience on negative coping style. Note: ^a^*p* < 0.001, ^b^*p* < 0.01, ^c^*p* < 0.05.

The results of APIMeM indicated that family resilience partially mediated the relationship between parenting stress and negative coping styles in parents (*β* = 0.207, *p* < 0.001), thus partially supporting the research hypothesis. Obviously, family resilience can buffer the impact of parenting stress on the likelihood of parents adopting negative coping styles. Moreover, the family resilience of parents played a completely mediating role in the relationship between parenting stress in parents and negative coping styles in grandparents (*β* = 0.169, *p* < 0.05), which also partially supported the research hypothesis (see Table 5 and Figure 3). The effect of grandparents' parenting stress on grandparents' negative coping styles is fully accounted for by the level of parents' family resilience.

## 4. Discussion

The results of this study indicated that family resilience partially mediated the relationship between parenting stress and positive coping styles in both parents and grandparents. Moreover, family resilience mediated partner effects, highlighting the interdependence between parents' and grandparents' stress and coping mechanisms. This demonstrated the dyadic associations among parenting stress, family resilience, and coping style in Chinese intergenerational co-parenting families.

Family systems theory and interdependence theory emphasize that the interdependence of family members is crucial for maintaining the balance of family function [15]. In intergenerational co-parenting families, parenting stress and coping styles interact between parents and grandparents [2]. In the context of intergenerational co-parenting culture in China, parents are responsible for the social upbringing of children, while grandparents, acting as “helpers,” are involved in aspects such as physical care-giving and family-related assistance. Due to differences in parenting values, paradigms, and sociocultural orientations, these two generations are prone to experiencing stress. Notably, the relationship between mother-in-law and daughter-in-law is particularly likely to encounter various parenting conflicts when jointly taking care of children [8]. Modern Chinese parents typically strive to foster their children's independence in daily life. They encourage kids to dress themselves, tidy their rooms, and manage personal matters on their own. This approach aims to cultivate self-reliance and problem-solving skills from an early age. Conversely, grandparents often shower their grandchildren with excessive love and care. They are more inclined to promptly satisfy their grandchildren's material and emotional desires [30]. Although the mother-in-law ostensibly acknowledges her daughter-in-law's dominant role in parenting, it is inevitable that she will surreptitiously manipulate the power dynamics to adjust their cooperative relationship [31]. Such conflicts prompt both parties to mobilize resources and employ diverse coping strategies to manage the resultant stress.

This study showed that both grandparents and parents had middle level of parenting stress, but parents was higher than that of grandparents, which is similar to previous studies [20]. The positive association between higher parenting stress and a more pronounced negative coping style means that when parents (or grandparents) experience greater stress in their parenting roles, they are more likely to adopt negative ways of dealing with that stress [32–34]. Negative coping styles might include avoidance (e.g., ignoring the problem) or excessive criticism and blame. This is particularly concerning because these coping approaches fail to address the root causes of stress and can exacerbate downstream challenges, such as declining mental well-being or strained interpersonal relationships. Our findings further indicated that family resilience is posited to act as a mediator, suggesting that family resilience may serve as a buffer against such stress [35]. A resilient family environment, characterized by strong bonds, effective communication, and a sense of shared purpose, may enable parents to better cope with stress in positive ways. For example, in a resilient family, members might support each other during difficult times, which in turn help parents maintain a positive outlook and use constructive coping mechanisms. Hu et al. conducted a study on 2635 grandparent caregivers and found that their parenting stress had a significant negative impact on family resilience [36]. However, the study also highlighted that the continuous interactive process of grandparents in providing support and solving problems played a crucial role in addressing adversity. Meanwhile, parents may leverage social or work networks and various resources to alleviate difficulties and adversity. However, grandparents often spend most of their time caring for grandchildren, thereby sacrificing social activities and interactions, which can erode their psychological resilience in daily life [37]. Notably, this study found that grandparents were more inclined to adopt negative coping strategies to manage stress, a finding that diverges from previous research [38]. A potential explanation is that, within the cultural context of China, grandparents often feel compelled to leave their homes and reside with their adult children following the birth of a grandchild. Most grandparents provide daily care for their grandchildren while relinquishing significant family decision-making authority, a dynamic that can foster feelings of powerlessness. Moreover, they may lack sufficient social support and meaningful communication channels [31, 38].

The APIMeM indicated that parenting stress had both actor and partner effects on positive coping in both parents and grandparents. In a family setting, the parenting stress experienced by one parent (the actor) can influence the family resilience and positive coping style of the other parent (the partner). Similarly, the family resilience demonstrated by one member can have an impact on the positive coping abilities of another [12, 39]. In a resilient family, parents and grandparents have a common goal of raising healthy children. This shared goal promotes mutual understanding and support. When one family member experiences parenting stress, others are more likely to offer practical help and emotional comfort, which in turn encourages the stressed individual to adopt positive coping strategies [40–42]. For example, if a parent is stressed about a child's behavioral problems, the other parent or grandparents may offer to take care of the child for a while to give the stressed parent a break, and also share their experiences and advice on dealing with such problems, facilitating the stressed parent's positive coping [35]. However, no partner effect of parenting stress was found on negative coping among grandparents. It might be because, although parents and grandparents may share certain common stressors concerning the child's well-being, the nature and intensity of these stressors can vary significantly depending on their distinct roles within the family. Each individual develops unique coping styles shaped by their personality traits, life experiences, and prior parenting encounters. These inherent individual differences in coping mechanisms may overshadow the potential partner effect, thereby reducing the likelihood that one family member's negative coping strategies will be directly influenced by another's stress levels [35, 43–45]. Furthermore, there are marked disparities in communication patterns between parents and grandparents. Parents often engage in highly frequent and intensive exchanges about parenting-related concerns [43, 45]. This continuous dialogue not only enables the transfer of stress but also significantly impacts their coping behaviors. Conversely, grandparents tend to have infrequent and superficial discussions regarding the stressors associated with grandparenting. The limited nature of these conversations means that the stress experienced by one grandparent rarely translates into a significant impact on the other's negative coping mechanisms. Additionally, grandparents often have a greater tendency to suppress and internalize their negative emotions related to grandparenting stress. Instead of openly sharing these feelings with their partners, they choose to deal with them privately. This self-containment further weakens the potential partner effect on negative coping, as the stress and associated negative emotions remain isolated within the individual rather than being shared and influencing the partner's responses. Considering the interaction between the parents and grandparents as a dyad, nurses should estimate the magnitude and significance of these actor and partner effects, providing a more comprehensive understanding of how these variables interact within families. This understanding can be crucial for developing interventions aimed at enhancing family well-being, reducing parenting stress, and promoting positive coping and family resilience.

### 4.1. Strengths and Limitations

This study employs the APIMeM to investigate the relationships among parenting stress, family resilience, and coping styles in Chinese intergenerational co-parenting households with children under 3 years old. Such research is relatively scarce within the field of family psychology. The findings not only offer valuable insights for enhancing the family resilience and stress-coping capabilities of intergenerational co-parenting families but also provide a solid foundation for developing targeted family care strategies.

Several limitations of this study should be acknowledged. Firstly, the cross-sectional design limits the ability to make causal inferences, and the use of self-report questionnaires introduces the possibility of response bias. Additionally, the convenience sample limits generalizability. Participants from one geographic area may not reflect the experiences of intergenerational co-parenting families elsewhere in China, especially for the distribution of monthly income within their population. A multicenter survey and longitudinal studies to examine how these dyadic relationships evolve over time or intervention studies aimed at building family resilience should be carried out for future study.

### 4.2. Implications for Policy and Practice

The present study offers novel family-based empirical evidence that can assist nurses in making informed decisions and implementing effective interventions for multigenerational households. Specifically, nurses should work with intergenerational families assess both parents' and grandparents' stress levels and offer interventions aimed at enhancing family resilience. Some potential interventions, such as communication skills workshops or family counseling programs, traditional festival culture adaptive intervention, digital support platform and community intergenerational parenting alliance, should be considered.

## 5. Conclusions

This study underscores the critical importance of focusing on family resilience as a pivotal element in promoting and sustaining intergenerational caregiving dynamics. Consequently, healthcare providers should implement targeted psychological interventions aimed at enhancing family resilience among parents. By doing so, these interventions can serve as a buffer against parenting stress, ultimately fostering more positive coping mechanisms in both parents and grandparents within the intergenerational caregiving context.

## Figures and Tables

**Table 1:** Sociodemographic characteristics of parents and grandparents (*N* = 312 pairs).

Variables	Parents (*n* = 312) *N* (%)	Grandparents (*n* = 312) *N* (%)
Age	31.72 ± 3.77	59.18 ± 2.96
Educational level		
Primary and below	24 (7.69%)	37 (11.86%)
Junior	15 (4.81%)	92 (29.49%)
Senior	22 (7.05%)	114 (36.54%)
College	129 (41.35%)	57 (18.27%)
University and above	122 (39.10%)	12 (3.84%)
Occupation		
Unemployed or part-time job	165 (52.88%)	230 (73.72%)
Full-time job	147 (47.12%)	82 (26.28%)
Monthly household income (Yuan)		
< 3000	55 (17.63%)	
3000–5000	144 (46.15%)	
> 5000	113 (36.22%)	
Parity		
A birth	238 (76.28%)	
Second child and above	74 (23.72%)	
Infant gender		
Male	169 (54.17%)	
Female	143 (45.83%)	
Child age (month)		
0 ∼ ≤ 12	101 (32.37%)	
12 ∼ ≤ 24	112 (35.90%)	
24 ∼ ≤ 36	99 (31.73%)	

**Table 2:** Comparison of parenting stress, family resilience, and coping style between parents and grandparents (*N* = 312 pairs).

	Parents	Grandparents	*t*	*p*
Parenting stress	35.21 ± 6.23	28.38 ± 7.94	19.766	< 0.001
Parental role satisfaction	15.86 ± 3.40	11.30 ± 4.32	25.818	< 0.001
Burden pressure	19.35 ± 3.85	17.08 ± 4.74	8.660	< 0.001
Family resilience	53.46 ± 6.31	51.64 ± 7.43	4.565	< 0.001
Responsibility	28.83 ± 3.72	27.63 ± 4.53	5.059	< 0.001
Control	9.72 ± 3.19	10.47 ± 2.72	−4.013	< 0.001
Challenge	14.91 ± 2.17	13.54 ± 2.38	10.476	< 0.001
Coping style	31.62 ± 7.13	30.21 ± 6.43	9.470	< 0.001
Positive coping	22.39 ± 5.05	19.89 ± 5.26	8.528	< 0.001
Negative coping	9.23 ± 2.55	10.32 ± 3.34	5.959	< 0.001

**Table 3:** The correlations among parenting stress, family resilience, and coping styles in parents and grandparents (*N* = 312 pairs).

Variables	1	2	3	4	5	6	7	8
*Parents (n = 312)*								
1. Parenting stress								
2. Family resilience	−0.636^∗∗^							
3. Positive coping	−0.549^∗∗^	0.512^∗∗^						
4. Negative coping	0.489^∗∗^	−0.542^∗∗^	−0.420^∗∗^					

*Grandparents (n = 312)*								
5. Parenting stress	0.653^∗∗^	−0.601^∗∗^	−0.446^∗∗^	0.465^∗∗^				
6. Family resilience	−0.417^∗∗^	0.482^∗∗^	0.302^∗∗^	−0.257^∗∗^	−0.463^∗∗^			
7. Positive coping	−0.433^∗∗^	0.461^∗∗^	0.496^∗∗^	−0.258^∗∗^	−0.503^∗∗^	0.454^∗∗^		
8. Negative coping	0.276^∗∗^	−0.450^∗∗^	−0.185^∗∗^	0.422^∗∗^	0.213^∗∗^	−0.284^∗∗^	−0.271^∗∗^	

^∗∗^ *p* < 0.01.

**Table 4:** The Actor-Partner Interdependence Mediation Model between parenting stress, family resilience, and positive coping style (*N = *312 pairs).

Path	β	SE	95% CI		*p*
Actor effects					
Parenting stress (parents) ⟶ positive coping style (parents)					
Total effect	−0.517	0.066	−0.670	–0.428	< 0.001
Direct effect	−0.394	0.059	−0.485	–0.202	< 0.001
Total indirect effect	−0.123	0.048	−0.247	–0.060	< 0.001
Parenting stress (parents) ⟶ family resilience (parents) ⟶ positive coping style (parents)	−0.121	0.051	−0.264	–0.060	< 0.001
Parenting stress (parents) ⟶ family resilience (grandparents) ⟶ positive coping style (parents)	0.002	0.019	−0.033	0.033	0.212
Parenting stress (grandparents) ⟶ positive coping style (grandparents)					
Total effect	−0.361	0.035	−0.476	–0.196	< 0.001
Direct effect	−0.244	0.049	−0.380	–0.111	< 0.001
Total indirect effect	−0.117	0.028	−0.180	–0.071	< 0.001
Parenting stress (grandparents) ⟶ family resilience (grandparents) ⟶ positive coping style (grandparents)	−0.073	0.027	−0.114	–0.011	< 0.001
Parenting stress (grandparents) ⟶ family resilience (parents) ⟶ positive coping style (grandparents)	−0.044	0.025	−0.130	–0.028	< 0.001

Partner effects					
Parenting stress (parents) ⟶ positive coping style (grandparents)					
Total effect	−0.137	0.040	−0.253	–0.093	< 0.001
Direct effect	−0.087	0.048	−0.187	0.002	0.067
Total indirect effect	−0.050	0.024	−0.110	–0.018	< 0.01
Parenting stress (parents) ⟶ family resilience (parents) ⟶ positive coping style (grandparents)	−0.074	0.022	−0.146	–0.004	< 0.001
Parenting stress (parents) ⟶ family resilience(grandparents) ⟶ positive coping style (grandparents)	−0.056	0.023	−0.135	–0.045	< 0.001
Parenting stress (grandparents) ⟶ positive coping style (parents)					
Total effect	−0.217	0.064	−0.342	–0.093	0.056
Direct effect	−0.062	0.048	−0.147	0.043	0.089
Total indirect effect	−0.155	0.053	−0.280	–0.066	0.030
Parenting stress (grandparents) ⟶ family resilience (parents) ⟶ positive coping style (parents)	−0.073	0.047	−0.132	–0.042	< 0.001
Parenting stress (grandparents) ⟶ family resilience (grandparents) ⟶ positive coping style (parents)	0.002	0.031	−0.125	0.001	0.132

**Table 5:** The Actor-Partner Interdependence Mediation model between parenting stress, family resilience, and negative coping style (*N = *312 pairs).

Path	β	SE	95% CI		*p*
Actor effects					
Parenting stress (parents) ⟶ negative coping style (parents)					
Total effect	0.399	0.066	0.221	0.451	< 0.001
Direct effect	0.207	0.057	0.064	0.348	< 0.001
Total indirect effect	0.192	0.044	0.052	0.387	< 0.034
Parenting stress (parents) ⟶ family resilience (parents) ⟶ negative coping style (parents)	0.176	0.035	0.013	0.267	< 0.001
Parenting stress (parents) ⟶ family resilience (grandparent) ⟶ negative coping style (parents)	0.015	0.055	−0.012	0.178	0.099
Parenting stress (grandparents) ⟶ negative coping style (grandparents)					
Total effect	0.416	0.025	0.231	0.634	< 0.001
Direct effect	0.288	0.042	0.023	0.436	< 0.001
Total indirect effect	0.128	0.038	0.016	0.373	< 0.001
Parenting stress (grandparents) ⟶ family resilience (grandparents) ⟶ negative e coping style (grandparents)	0.026	0.073	−0.145	2.023	0.523
Parenting stress (grandparents) ⟶ family resilience (parents) ⟶ negative coping style (grandparents)	0.129	0.071	0.034	0.259	< 0.001

Partner effects					
Parenting stress (parents) ⟶ negative coping style (grandparents)					
Total effect	0.268	0.043	0.121	0.396	0.021
Direct effect	0.019	0.058	−0.127	0.154	0.241
Total indirect effect	0.249	0.022	0.092	0.366	0.042
Parenting stress (parents) ⟶ family resilience (parents) ⟶ negative coping style (grandparents)	0.106	0.032	0.003	0.234	< 0.001
Parenting stress (parents) ⟶ family resilience (grandparents) ⟶ negative coping style (grandparents)	0.020	0.017	−0.012	0.126	0.193
Parenting stress (grandparents) ⟶ negative coping style (parents)					
Total effect	0.387	0.054	0.186	0.545	0.039
Direct effect	0.142	0.048	0.021	0.239	0.021
Total indirect effect	0.245	0.053	0.019	0.371	0.036
Parenting stress (grandparents) ⟶ family resilience (parents) ⟶ negative coping style (parents)	0.169	0.023	0.089	0.254	0.012
Parenting stress (grandparents) ⟶ family resilience (grandparents) ⟶ negative coping style (parents)	0.019	0.036	−0.041	0.188	0.340

## Data Availability

The data that support the findings of this study are available from the corresponding authors upon reasonable request.
